# The complete mitochondrial genome of Hine’s emerald dragonfly (*Somatochlora hineana* Williamson) via NGS sequencing

**DOI:** 10.1080/23802359.2018.1463824

**Published:** 2018-05-10

**Authors:** Craig Jackson, Sunnie Grace McCalla, Jon Amberg, Dan Soluk, Hugh Britten

**Affiliations:** a U.S. Geological Survey, Upper Midwest Environmental Sciences Center, La Crosse, WI, USA;; b Department of Biology, University of South Dakota, Vermillion, SD, USA

**Keywords:** Hine’s emerald dragonfly, mitogenome, *Somatochlora hineana*, Odonata

## Abstract

Here, we report the complete mitochondrial genome of the endangered Hine’s emerald dragonfly (HED), *Somatochlora hineana* Williamson. Data were generated via next generation sequencing (NGS) and assembled using a mitochondrial baiting and iterative mapping approach. The full length circular genome is 15,705 bp with 26.6% GC content. It contains the typical metazoan set of 37 genes: 13 protein-coding genes, 22 transfer RNA (tRNA) and 2 ribosomal RNA (rRNA) genes, and an A + T-rich control region. To our knowledge, this is the first report of the complete HED mitogenome.

The Hine’s emerald dragonfly, *Somatochlora hineana* Williamson (HED), was listed as endangered by the USFWS in 1995. HED currently occurs in Wisconsin, Missouri, Illinois, Michigan, and Ontario, Canada. It has been extirpated from Ohio and probably Indiana. Much HED habitat has been lost to agricultural, urban, and industrial development, which alter the quality and quantity of groundwater flowing into the calcareous fens required by the larvae. Pesticides, roadway development and groundwater contamination also pose significant ongoing threats to HED. Management and conservation efforts based on traditional species monitoring methods are costly and laborious, requiring invasive sampling. The development of a molecular-based assay for the specific detection of HED mitochondrial DNA from the environment (i.e. water samples) would overcome these limitations and lead to more accurate and timely decisions by resource managers who are working to preserve and protect HED habitat. The first step in developing such an assay is to obtain and compare the HED mitogenome to that of closely related species.

Genomic DNA was extracted from leg tissue of the three adult HED specimens. Two specimens were collected in Door County, WI (Specimen ID 18428, collected at 44°49′15.44″N, 87°17′25.88″W; Specimen ID 23535, collected at 45°7′59.80″N, 87°6’33.66”W) and one specimen was collected in Will County, IL, USA (Specimen ID 11818, collected at 41°35′5.60″N, 88°4′53.52″W). Voucher specimens reside at the University of South Dakota Museum Storage. Library preparation was performed using the Illumina Nextera XT kit and whole genome sequencing was done on the Illumina NextSeq-500 platform using a Mid Output v2 kit to produce 150bp paired-end reads (Illumina, San Diego, CA, USA. The mitogenome was reconstructed using MITObim version 1.9 (Hahn et al. 2013) using a partial mitochondrial gene sequence for *Somatochlora williamsoni* cytochrome oxidase subunit 1 (KM531663.1) as the reference seed. This assembly was aligned with the mitogenomes of 25 other insects of subclass *Pterygota* using Geneious v10.2.2 software (http://www.geneious.com/). The MITOS WebServer (mitos.bioinf.uni-leipzig.de/index.py) was employed to add annotations, which were confirmed by manual BLAST search (Altschul et al. 1990). The phylogenetic relationship of *S. hineana* Williamson to several related odonate mitogenomes is presented in Figure 1.

The complete circular HED mitogenome sequence (MG594801) is 15,705 bp in length and contains the typical metazoan set of 37 genes: 13 protein-coding genes, 22 tRNA and 2 rRNA genes, and an A + T-rich control region (85.9% A + T). The gene arrangement is identical to that of other odonate mitogenomes (Simon and Hadrys 2013; Yu et al. 2014) with the exception of a 515 bp region located between *trnT* and *trnP*, which appears to contain duplications of these genes and a partial duplication of *nad4l*. Four of five standard invertebrate mitochondrial start codons were found: TTG (*cox1*), ATT (*nad2*,* atp8*,* nad5*,* nad1*), ATA (*nad3*, *nad4l*, *nad6*), and ATG (*cox2*, *atp6*, *cox3*, *nad4*, *1cob*). No genes with start codon ATC were identified. All protein-coding genes use the stop codon TAA except *nad3* and *nad1*, which use TAG. The tRNA genes range from 62 bp to 72 bp. Three intergenic spacer regions were found, similar to those described in other odonates such as *Anax imperator* (Herzog et al. 2016).[Fig F0001]

**Figure 1. F0001:**
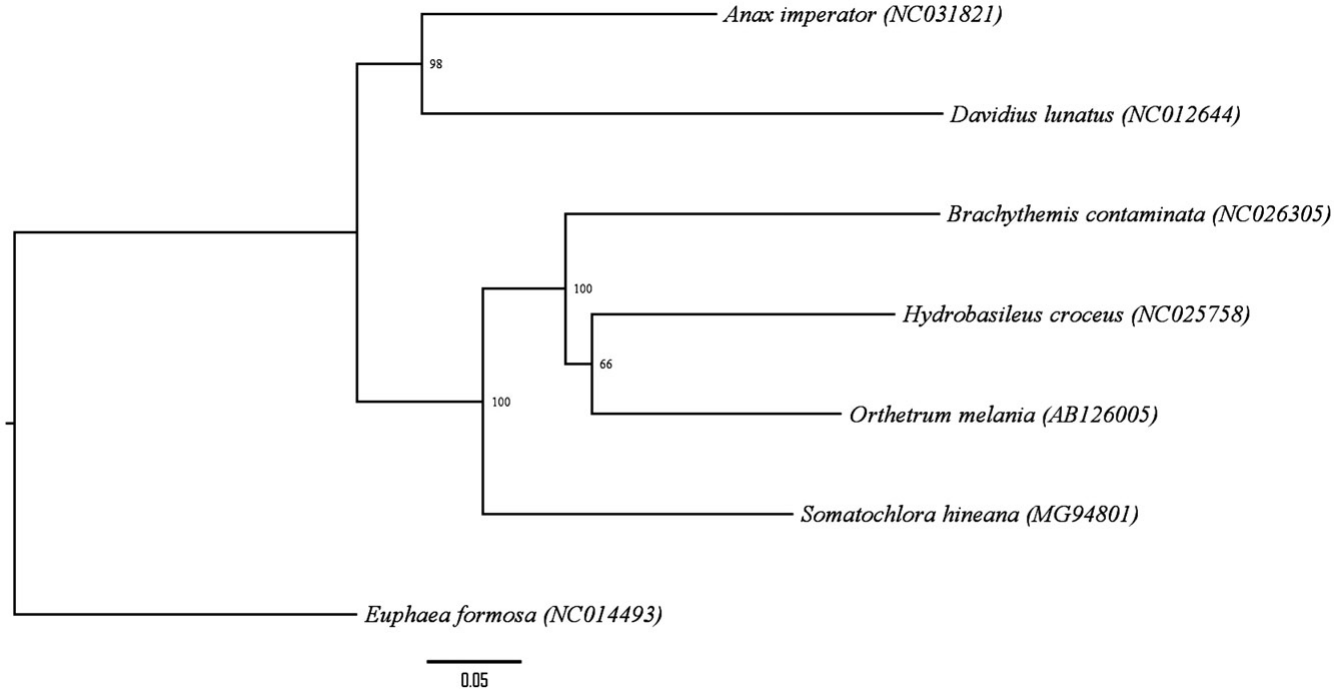
Maximum-likelihood (RAxML) phylogeny using *Euphaea formosa* as an outgroup. Node labels are support values from 1000 bootstrap replicates. Annotation was performed using FigTree v1.4.3.
